# Introducing Public Health Vending Machines in Rural Communities: Protocol for a Study Using a Community-Based Participatory Approach

**DOI:** 10.2196/64913

**Published:** 2025-09-17

**Authors:** Meghan Guter, Lauren Harrell, Kathleen L Egan, Reese Hiatt, Lori Ann Eldridge

**Affiliations:** 1 Department of Health and Human Performance East Carolina University Greenville, NC United States; 2 Department of Implementation Science Wake Forest University School of Medicine Winston-Salem, NC United States

**Keywords:** public health vending machine, naloxone, community-based participatory approach, rural, protocol, North Carolina, community-based participatory research, CBPR

## Abstract

**Background:**

Drug-related overdoses impact communities all over the United States. In the past 2 decades, over 28,000 people have died of a drug overdose in North Carolina (NC). Research has shown that there has been an increase in overdose deaths throughout NC, particularly in rural areas. To reduce overdose rates, health care interventions should be expanded. Naloxone distribution is one intervention to combat overdose rates. Naloxone is a medication designed to reverse an opioid overdose rapidly. Public health vending machines (PHVMs) are a strategy recently implemented in some US communities to expand access to harm reduction supplies. Examples of locations where PHVMs have been installed include public health departments, libraries, county detention centers, and law enforcement offices.

**Objective:**

This protocol aims to develop a community-engaged approach to implementing PHVMs as a health care delivery option for harm reduction supplies in 5 rural counties in NC.

**Methods:**

This study will use a community-based participatory approach in which we partnered with the NC Harm Reduction Coalition and Community Impact NC to engage with substance use prevention providers and community members in 5 rural counties in NC to improve naloxone access. We will collect qualitative interview data from people with lived experience of substance use to identify the optimal placement of PHVMs and items to be stocked in PHVMs. To do this, we will hire 1 local community member with lived experience of substance use from each county to be an interviewer who will recruit, conduct interviews, and collect data from other community members with lived experience of substance use. Interviewers will be trained to recruit participants, conduct interviews, and collect and analyze data. Developing a protocol for training interviewers includes an interview training presentation with an adapted collaborative institutional training initiative portion.

**Results:**

Data will be collected from 2024 to 2025. The findings will inform the implementation of PHVMs to improve harm reduction access and assist in decreasing overdose deaths.

**Conclusions:**

This study will use in a community-based participatory approach to improve naloxone access in rural communities. Community partners will assist the academic team in developing a sustainability plan for each county and an implementation toolkit for other communities to use.

**International Registered Report Identifier (IRRID):**

PRR1-10.2196/64913

## Introduction

### Overdose Epidemic in the United States

In the United States, there is a growing epidemic of drug-related overdoses. Since 1999, there has been a continuous increase in the number of drug-involved overdose deaths in the country [1]. In 2022, there were 105,452 drug overdose deaths [2]. This epidemic affects all genders, ages, and racial and ethnic groups. The number of deaths from drug overdoses has increased drastically, particularly among individuals living in rural areas throughout the country [3]. In studies examining where overdose deaths occurred, it was found that rural areas had the highest rates of drug overdose deaths [1].

Recently, the United States has experienced a notable decline in drug overdose deaths, marking a significant shift in the trajectory of the overdose crisis. Provisional data from the Centers for Disease Control and Prevention indicate a 3% decrease in deaths in 2023, which was the first annual decline since 2018 [4]. This downward trend has continued into 2024, with preliminary figures showing nearly a 24% decline from the previous year and the lowest total since June 2020 [5]. Despite these encouraging developments, the overdose crisis remains complex. While deaths involving opioids, particularly synthetic opioids such as fentanyl, have decreased, fatalities associated with stimulants such as cocaine and methamphetamine have risen [6]. In 2023, overdose deaths involving cocaine increased by 4.9%, and those involving psychostimulants rose by 1.9% [7]. Moreover, polysubstance use—especially combinations of opioids with stimulants—continues to drive a significant portion of overdose deaths (nearly 50% in 2021), underscoring the evolving nature of the epidemic [8].

The North Carolina (NC) age-adjusted drug overdose mortality rate almost doubles the national average according to the most recent data published by the Centers for Disease Control and Prevention [9]. The combined cost of opioid use disorder and fatal opioid overdose in NC was US $39.37 million; NC ranked eighth in the United States based on the high economic toll of the opioid crisis [9]. In total, 40% of NC residents live in a rural county according to the NC Department of Commerce [10]. The NC Department of Commerce identifies that 80 out of the 100 counties that make up NC are rural based on a population density of 250 people per square mile or less [10].

Over the past decade, in NC, rural populations have experienced higher overdose mortality rates than residents of metropolitan areas [11]. Rural areas comprise open country and settlements with under 5000 residents and under 2000 housing units [12]. Rural communities in NC have higher rates of drug and alcohol use, suicide, years of productive life lost, injury, uninsured patients, and preventable hospitalizations. Residents of rural areas in NC face a shortage of all types of medical providers [13]. The higher rates and shortages associated with rural areas affect the communities’ overall health outcomes. These disparities are shaped by longstanding social and structural factors that limit access to health care and infrastructure development and foster economic disinvestment and historical marginalization [14].

The expansion of naloxone access has been a priority intervention to combat the overdose epidemic. Naloxone is a fast-acting opioid antagonist medication that, when administered promptly, can reverse an overdose from opioids—including heroin, fentanyl, and prescription opioid medications [15]. Naloxone is easy to use, and there are 2 forms that anyone can use without medical training: prefilled nasal spray and injectable [15]. For naloxone to be effective, it must be made available and used within the correct time frame.

### Increasing Availability of Naloxone

To reduce opioid overdose deaths, organizations have worked to make naloxone more readily available and easily accessible to lay individuals to assist with combating the rising rates of overdoses; as of 2023, naloxone has become available to the public in all 50 states as an over-the-counter medication [16]. Lay individuals can access naloxone through their local pharmacy, community-based naloxone programs, most syringe services programs (SSPs), and local health departments [17]. The availability of naloxone has been shown to have an impact on decreasing rates of drug overdose deaths [17]. Studies have shown that, in locations where there was more accessible naloxone, the rates of drug overdose were lower than in locations with less availability [18].

In recent years, public health vending machines (PHVMs) have been installed in various locations to help increase access to naloxone and other wellness supplies. Supplies offered in machines can change over time and include various items such as over-the-counter medications (paracetamol, antihistamines, and antacids), sunscreen, insect repellent, socks, water, syringes, clean smoking supplies, testing strips, hand warmers, dental kits, hygiene items, and diapers. PHVMs allow access to life-saving supplies that might otherwise have been difficult for one to access [19]. The Trac-B Exchange program in Nevada installed PHVMs in a family medical clinic, behavior health offices, gay and lesbian community centers, substance use treatment centers, a health district office, and a detention center to examine the extent to which naloxone dispensation in PHVMs was associated with changes in overdose fatalities [20]. They found that, within a year following the installation of the PHVMs, naloxone dispensation in PHVMs was associated with immediate reductions in overdose fatalities [20]. In New York, the Medication for Addiction Treatment and Electronic Referrals network deployed 15 harm reduction vending machines stocked with Narcan and fentanyl and xylazine test strips across the state [21]. All supplies from the vending machines are accessible at no cost [22]. The new service was implemented to allow individuals and organizations across the state to request harm reduction supplies free of charge [22]. In Cincinnati, Ohio, the implementation of an automated harm reduction dispensing machine led to increased accessibility of harm reduction products and services [23]. It was associated with a lower countywide incidence of unintentional overdose death and HIV [3]. In Los Angeles County, California, naloxone vending machines were introduced in public spaces at detention centers [8]. After the first 9 months, the machines reported distributing >20,000 doses of naloxone. Several other jurisdictions nationwide have followed this example and expanded naloxone access in detention centers [24]. In NC, naloxone vending machines have been implemented in detention centers in the Buncombe, Cumberland, Forsyth, Guilford, Orange, Pitt, and Wilkes counties at no cost through a program sponsored by the National Center for State Courts [25]. The NC Harm Reduction Coalition (NCHRC) assisted in identifying sheriffs and officials to help distribute naloxone, find optimal placement for the machines in facilities, and assign responsibility for filling the machines [26]. Some research suggests that younger people and female individuals tend to use PHVMs more frequently to avoid stigma from other facilities where one could obtain harm reduction supplies, such as an SSP [27]. There are barriers to PHVM implementation, for example, some communities’ attitudes (stigma) and fear of vandalism or the fact that all the supplies will be depleted by 1 person [28,29]. Other factors that contribute to barriers are that people who use drugs choose not to use PHVMs due to police intervention located near them [26]. Additional barriers are related to the lack of planning for the placement of the machines or the need to register for the machine to use it [30].

### Lack of PHVMs in Rural Areas

PHVMs have been associated with decreasing rates of drug overdoses [31]. Most PHVMs have been implemented in urban or suburban areas, and there has been very little evidence of implementation in rural areas [32]. Participants in these studies have demonstrated a desire to have naloxone PHVMs available in rural areas to reach underserved communities [33]. There is a clear gap in current research concerning expanding PHVMs to hard-to-reach locations with limited substance use resources. Future work should be conducted to better understand how to scale up overdose prevention interventions, including PHVMs, in rural areas. Each community has unique needs and cultures, which would benefit from a tailored approach to implementing PHVMs. Thus, there is a need for a protocol to help communities identify their unique needs for overdose prevention to establish PHVMs in those locations. Furthermore, studies on the sustainability of PHVMs have been limited. Due to the novelty of PHVMs, there are limited data on their long-term impact and sustainability.

### Objectives

This study’s purpose is to use a community-based participatory approach to improve naloxone access in rural communities in NC. The results will be used to improve operational procedures for PHVMs.

## Methods

### Community-Based Participatory Approach

This study uses a community-based participatory research (CBPR) approach. A CBPR approach is a collaborative research approach that will involve community members, researchers, and other stakeholders in the research process, combining knowledge and recognizing the strengths that each entity can bring [34]. CBPR will provide a framework to equitably involve all stakeholders of the research, identifying and maximizing the importance of their diverse contributions [35]. CBPR has been used to help understand health disparities and promote health equity across diverse populations and issues that matter to communities of concern [36]. CBPR will be a vital strategy for engaging communities in research directly affecting their lives. Our study team will use the Structural Indicators for Community-Based Participatory Action Research to guide their work [37]. It is essential to our study team to establish meaningful engagement and partnerships with people who use drugs in our selected communities. These relationships will be essential to conducting research that will be impactful in supporting the desired implementation outcomes and reducing drug-related harms [38].

**Figure 1:**
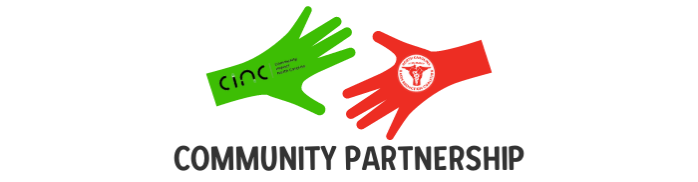
Community partnership consisting of North Carolina Harm Reduction Coalition, Community Impact North Carolina, community members, and community organizations.

**Figure 2:**
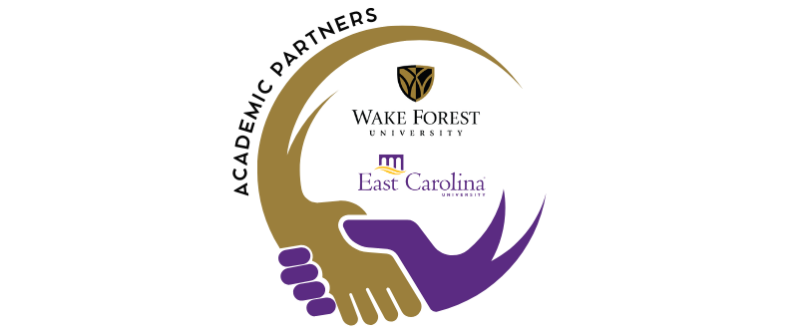
Academic partners: East Carolina University and Wake Forest University.

Our partnership consists of academic partners (East Carolina University and Wake Forest University School of Medicine), the NCHRC, Community Impact NC (CINC), and community members and community organizations (Figures 1 and 2) located in 5 rural counties in NC. The academic partners have an established working relationship and a shared history of engaging in community outreach and research focused on substance use. Their collaboration is strengthened by mutual experience and a commitment to addressing substance use challenges in meaningful, community-driven ways. The community partners, the NCHRC and CINC, are key collaborators in this work and currently oversee an overdose prevention grant. As part of this grant, they provide support and oversight to awardees across the state. Notably, the 5 counties where we propose to implement the PHVM initiative are all recipients of this grant funding, positioning them well for successful implementation and ongoing partnership.

Through collaborative efforts with our community partners (CINC and the NCHRC) and individuals with lived experience with substance use from the NCHRC, the team cocreated all training and study materials. This will involve an iterative process in which materials will be reviewed, feedback will be given, and revisions will be made. This process will be repeated until the partnership agrees that the materials meet the standards of all members. By applying insights and expertise from our partners, we will tailor the materials to resonate most effectively with our target communities and participants.

### Ethical Considerations

The expedited application for this study (University and Medical Center Institutional Review Board 23-001800) was approved on September 11, 2023, by the corresponding authors’ institution.

### Setting

**Figure 3:**
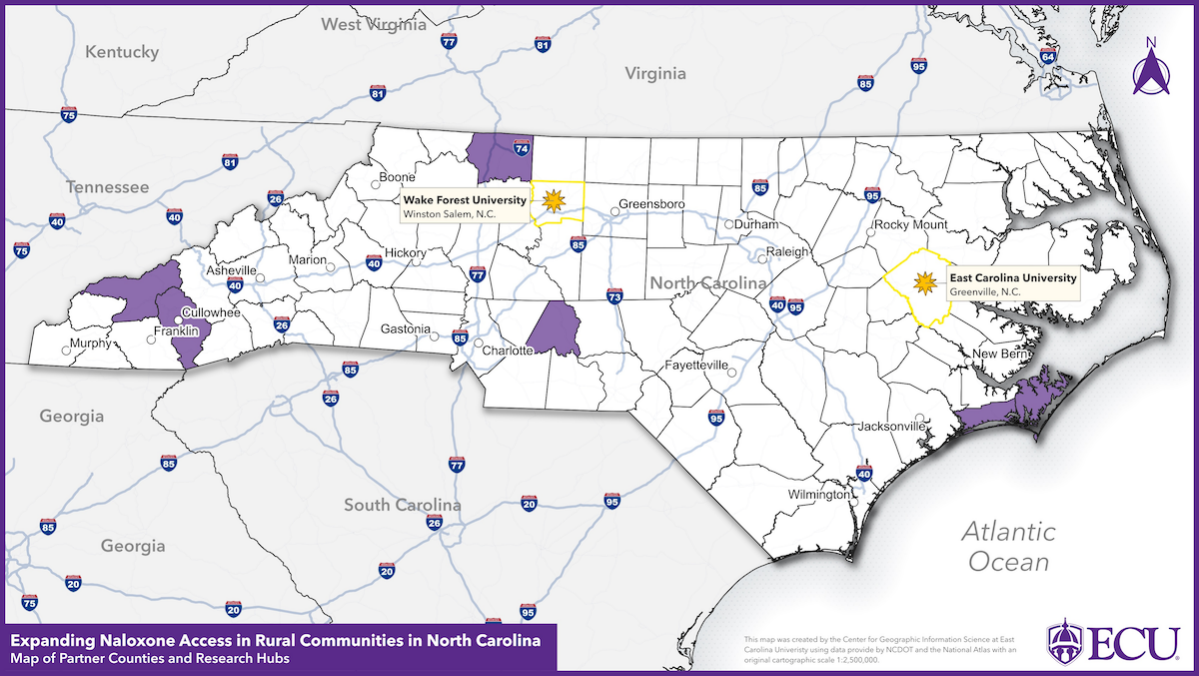
Map of partner counties and research hubs.

Five counties in NC (Figure 3; Carteret, Jackson, Surry, Stanly, and Swain) were selected based on the following criteria: (1) being a Health Resources and Services Administration–designated rural county, (2) high rates of drug overdose deaths, (3) high rates of illicit opioid–involved overdose deaths, (4) high rates of drug overdose emergency department visits, (5) high rates of residents receiving an opioid prescription, and (6) having an established relationship with CINC. The 5 counties are modest in size, represent different regions of NC, and have structural inequities. Furthermore, all 5 counties have documented drug overdose disparities based on the race and ethnicity of the residents, with higher rates than the state of overdoses among American Indian, Alaska Native, or Black non-Hispanic residents [13]. In NC, naloxone is primarily distributed to laypersons by SSPs and medical providers such as pharmacies. There are currently 71 registered SSPs that serve residents of NC [26]. However, only 2 of the 5 participating counties are currently served by an SSP, and the SSPs that serve the 2 counties are not physically located in the counties. There is a demonstrated need to expand naloxone distribution within these 5 counties by establishing a fixed-care delivery site.

### Interview Guide

The interview guide (Multimedia Appendices 1-3) was developed in direct collaboration with our community partner, who brings valuable lived experience with substance use. The questions in the guide were shaped by the stakeholders’ priorities and insights, the literature, and grant deliverables (establishing a care delivery site [PHVM or other mechanism], improving access to naloxone, determining the preferred placement of PHVMs, publicizing the PHVMs, and assisting with developing a county-specific sustainability plan).

### Identifying and Hiring Community Members to Serve as Interviewers

Interviews with local community members (n=10 per county) will be conducted to identify optimal placement for implementing a PHVM stocked with naloxone. The academic team, in collaboration with local community partner organizations, the NCHRC and CINC, will take the lead in identifying and recruiting community members to serve as interviewers. As a first step, the academic team will participate in video conference town hall meetings that will be hosted by CINC and the NCHRC where they will engage with substance use prevention providers from each community. Following these town hall meetings, the academic team will meet individually with each prevention provider or their designated community representative to further discuss and recruit potential interviewers. CINC and the NCHRC will coordinate these meetings and be present to support the process.

To facilitate outreach, the academic team will request names and contact information for local organizations that work with individuals with lived experience. These organizations may include peer recovery groups, faith-based health initiatives, local health departments, re-entry programs, and postoverdose response teams. To support this effort, academic partners will ask local community partners to provide a “warm handoff” by introducing them to these organizations through email. This personal introduction will help establish trust and assist with effective interviewer recruitment.

Individuals eligible to participate as interviewers must be aged ≥18 years and live in 1 of the 5 selected counties: Carteret, Jackson, Surry, Swain, and Stanly. They will have lived experience of using substances such as opioids, fentanyl, cocaine, or psychostimulants; work within the harm reduction agency (paid or volunteer); and be able to provide references of their experience and qualifications.

After the meeting, the academic team will connect with community partners to discuss potential candidates for interviewer roles. To ensure that selected individuals meet the necessary criteria, the academic team will ask those recommended to complete an application. This application will provide details about their experience conducting interviews; any involvement in research, whether as a participant, a researcher, or both; the recency of their lived experience; their level of community involvement; their current work with harm reduction agencies; and any other relevant experience. Before making final hiring decisions, the academic team will meet individually with each candidate. Throughout this process, the partnership will collaborate closely with local community partners to identify and select individuals best suited for the interviewer positions.

Once interviewers are recruited and hired, they will be trained through a video conference presentation conducted by the academic team. The interviewers will be classified as consultants and paid US $1000 for their work. The payment will occur upon completion of the 10 interviews.

### Training Community Members to Serve as Interviewers

**Figure 4:**
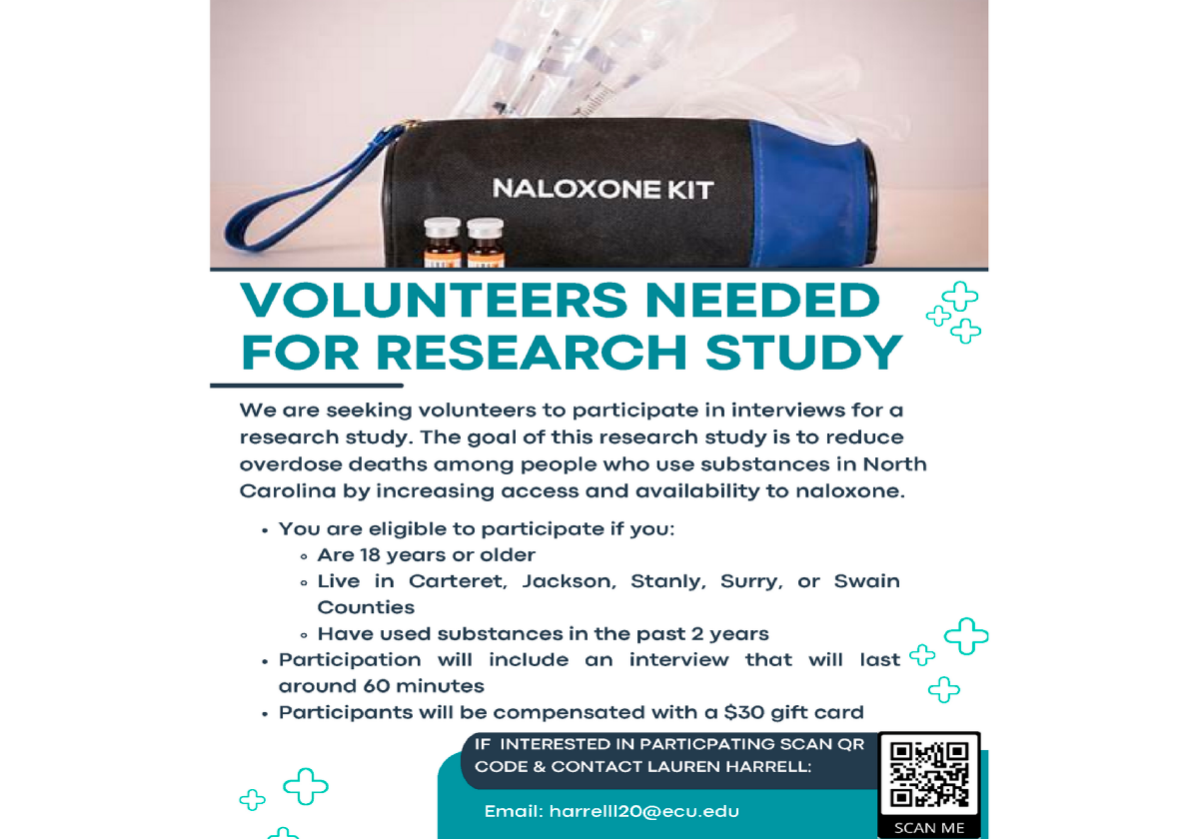
Recruitment flyer seeking volunteers to participate in the interviews.

**Figure 5:**
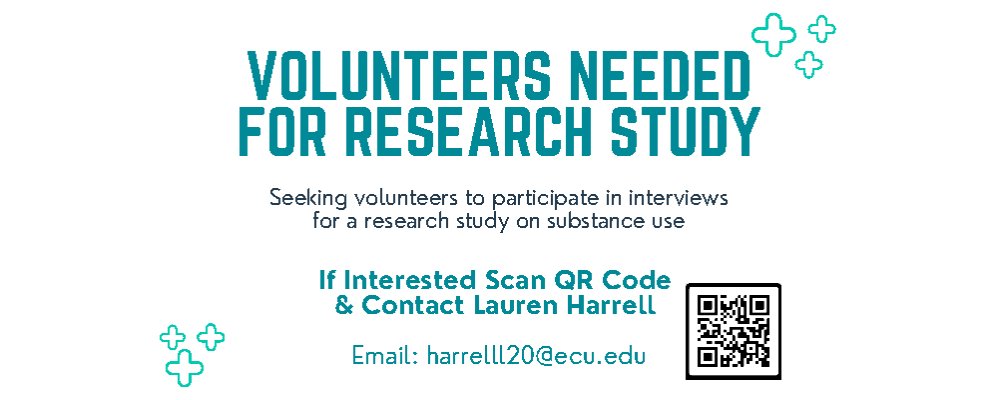
Recruitment pass-along card surveyors share to recruit individuals to participate in the interviews (front).

**Figure 6:**
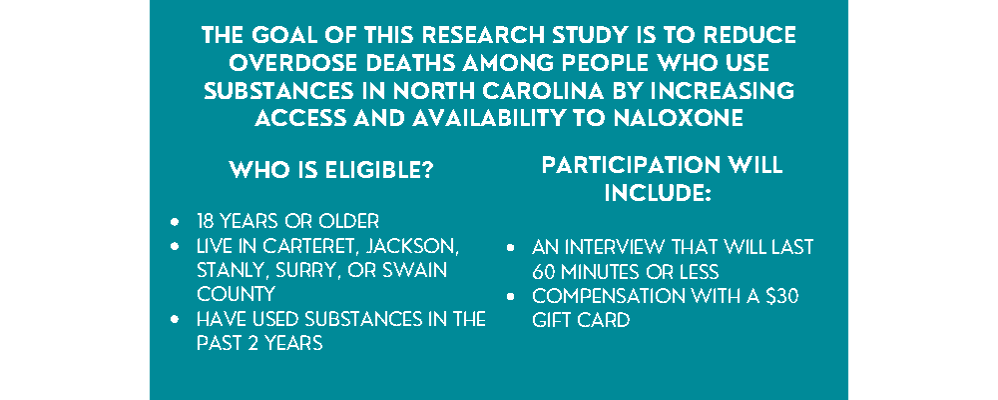
Recruitment pass-along card surveyors share to recruit individuals to participate in the interviews (back).

The training protocol for interviewers will include a virtual interview training presentation (Table 1) with an adapted collaborative institutional training initiative portion. The training presentation includes how to conduct interviews, analyze the data, and share the findings from the interviews. Interviewers will be encouraged to recruit participants through personal contacts and given tools to help recruit. These tools include a recruitment script (Multimedia Appendix 4), flyers (Figure 4), and pass-along cards (Figures 5 and 6). The tools will be mailed to interviewers before the training so they can be reviewed during the training.

**Table 1:** Interview training presentation outline.

Section	Description of section
Introduction to the study and purpose of training	Introduction to the study team, objectives of the study, overview of agenda, and review of materials
CITI^a^ training	Introduction to the key principles and ethical considerations involved in human subject research as covered by the CITI program; a simplified—user-friendly—CITI training was developed by the partnership and approved by the IRB^b^
Conducting interviews	Steps to conduct the interview, how to schedule and recruit people to interview, how to obtain participant consent, learning how to probe and use an interview script, note taking and documenting the interview, and distribution of reimbursement to participants
Safety	How to end an interview if they are feeling unsafe and determining a safe location for the interview to take place
After the interview	How to contact and follow up with the study team
Team data analysis	Data collection and how to analyze qualitative data
Share findings	Data reporting and poststudy involvement

^a^CITI: Collaborative Institutional Training Initiative.

^b^IRB: institutional review board.

### Interview Participants

To be interviewed for this project, participants must reside in 1 of the 5 preidentified high-need rural counties in NC. Participants must be aged >18 years and have lived experience of using substances such as opioids, fentanyl, cocaine, or psychostimulants in the past 2 years. No one is excluded from this study based on race, ethnicity, or biological sex. A total of 10 interviews will be conducted in each county, totaling 50 interviews, which aligns with commonly accepted thresholds for achieving data saturation in qualitative research [39]. This sample size is sufficient to capture a range of perspectives and identify recurring themes without the emergence of substantially new information in later interviews.

### Interview Recruitment

Interviewers will be provided with a list of names of interested community members by the study team. Interviewers will recruit participants using word of mouth, a recruitment script, recruitment flyers, and pass-along cards provided by the academic team. Interviewers will be encouraged to share information with personal contacts to help recruit participants. Interviewers will notify the academic team of participants before conducting the interviews. Interviewers will schedule the interviews by contacting the participants based on the information given by the study team. Each interviewer will conduct 10 interviews per county. Interviewers will be encouraged to engage a diverse range of participants, including individuals of different sexes, races, and ethnicities. They will also be guided to conduct interviews across various areas of their county rather than focusing solely on 1 neighborhood or the county seat to ensure broader geographic representation.

### Interview Data Collection

The location of the interview will be determined through a reference interview location list that will be established with the assistance of the partnership. The location will be comfortable and safe for both the participant and the interviewer. The principal investigator or coinvestigator of the study team will conduct a follow-up meeting with the interviewer 72 hours following the completion of an interview. During this follow-up, the interviewer can ask questions, seek clarification, and receive additional training as needed. In addition, this time will be used to discuss the thoughts or notes taken during the interview. This step is very important in case the interview questions or procedure need to be adjusted.

All participants’ personal information will remain confidential. During the interviewer training, interviewers will be trained on how to protect participants’ privacy and obtain consent. Before starting the interviews, interviewers will provide participants with an alias.

**Figure 7:**
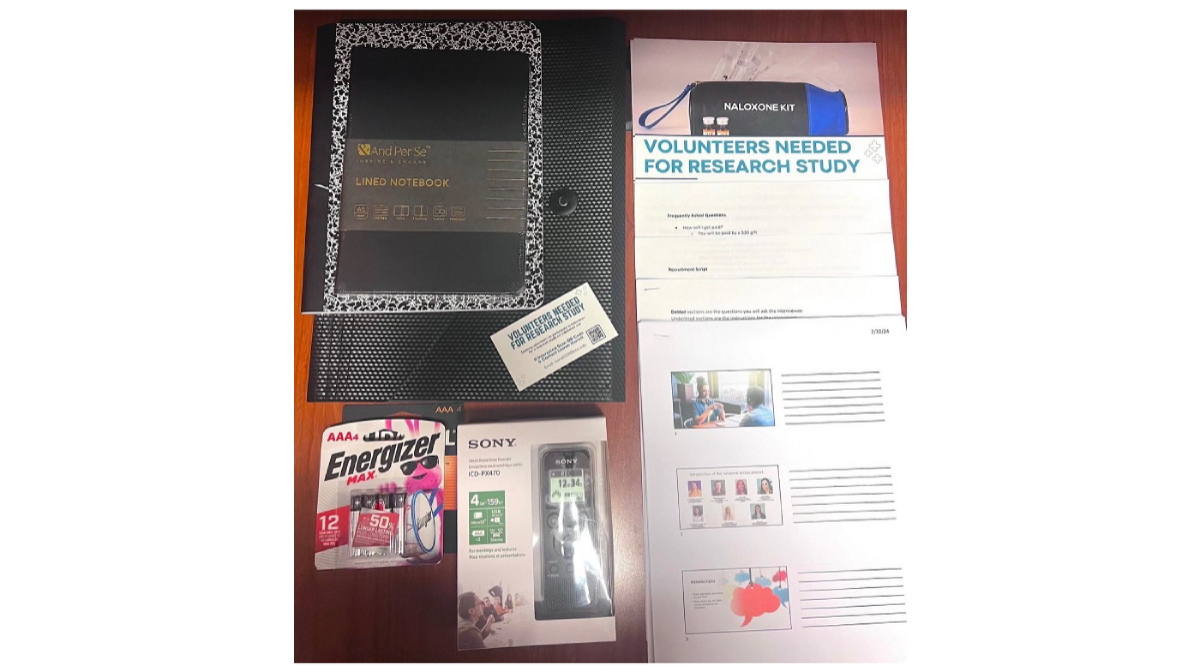
Surveyor interview toolkit. These items are provided to surveyors before training and recruitment of participants.

During the training, before the interviews, interviewers will be provided with interview kits containing a recorder, notebook, batteries, frequently asked question answer sheet, acknowledgment form, and interview script (Figure 7). The interview script (Multimedia Appendix 4) will provide interviewers with questions to ask and follow during the interview.

First, interviewers will provide the participant with a copy of the consent form and will read the consent form to the participant. The interviewer will inform the participant that they may take notes if they ask any questions that were not on the interview guide or if they have a thought that they want to write down to share with the academic team. The interviewer will tell the interviewee that they have the right to look at the notes. After the participant has agreed to take part, the interview will start. At the start of the interview, interviewers will turn on the recorder and state the participant’s alias and the date and county. The interviewer will have the participant state the following: “I consent to this interview.”

After the interviewer has completed the interview, they will encourage the participant to recruit other participants and provide them with 5 pass-along cards (Figures 5 and 6). The interviewer will then distribute a gift card for US $35 to the participant. The interviewer will record the participant saying that they received the gift card, have the participant sign the acknowledgment form with their alias, and document in their notebook that they provided the gift card with the date and alias name. To conclude the interview, the interviewer will thank the participant and turn off the recorder. Upon completing the 10 interviews, the interviewer will return the recorder and acknowledgment sheet to the study team via a prepaid envelope.

### Interview Data Analysis

After concluding the interviews, the interviewer will provide their data to the academic team. They will also provide their notes from the interviews, all documents that the participants signed, and the recordings. The academic partners will be responsible for data management, analyses, and dissemination. The academic partners will transcribe the recordings of the interviews and organize the data using NVivo (QSR International). The interviewers will be asked to join the academic team meetings to assist in interpreting the data together.

The qualitative data will be coded using a priori codes and emergent themes. The a priori codes will be that rural communities face significant barriers in obtaining naloxone, including stigma and limited availability [31]. Two people from the academic partnership will independently code the sample to achieve a uniform understanding of the codes. A coding conference will be held to identify and manage any code discrepancies. Upon agreement on the final coding scheme, a conference will be held with all interviewers (n=5) and the partnership to review the codes and confirm observations and findings. If any discrepancies arise, they will be discussed and addressed during this meeting. The final coding scheme will then be used by a primary coder from the academic partners to code all interviews. A final data conference will be held with the entire partnership to confirm observations and determine priority findings related to the operational procedures for PHVMs, location, and items to be stocked [39-41].

### Review of Interview Findings With Community Decision-Makers

The academic partners will work hand in hand with the community partners to identify key individuals within the county who are decision-makers as well as early adopters of the PHVMs. Rogers [42] states that early adopters are people or organizations willing to implement the “new practice” before the public. Decision-makers may include law enforcement, emergency management services, faith-based leaders, prevention and treatment providers, public health departments, hospital personnel, and other advocates. Early adopters will be vital in assisting the partnership in creating opportunities for relationships within each rural community. In addition, early adopters can advocate for the PHVM among the county residents and other key decision-makers. They act as a conduit to provide vital information about the community to the partnership and vice versa. Such information includes details about the county’s unique region, including its history, institutions, traditions, and demographics. Understanding these characteristics within rural communities has been suggested as a way to create capacity and enable implementation projects that will be sustained over time [38]. The partnership, along with early adopters and the interviewer, will collaboratively identify individuals and organizations in each community to disseminate the findings. In addition, they will identify how and where to share the findings with the community. Securing support from influential community members (elected officials) is necessary [38]. Thus, the partnership will collaborate closely with early adopters to identify decision-makers and elected officials. Studies indicate that rural implementation success is strengthened with relationships; thus, having an advocate such as the early adopter attend in-person meetings is important [43]. Community engagement strategies in rural settings are different from those in urban settings, and it has been shown that increasing participation, especially of all community members, within rural communities leads to improved sustainability [43]. Therefore, an intentional selection of key individuals is necessary.

Upon completion of the identification of key community members, an email will be sent by the partnership introducing the team and the project and inviting them to attend an informational meeting. The early adopter may also send an email or call individuals and provide a personal invitation to the meeting. This step may be vital in communities where there are critics who could prevent the successful implementation or sustainability of the PHVM. Studies have found that credibility in rural communities is developed from the connection to the community rather than from an institution, degree, or title [38].

The structure of the informational meeting will be determined by the partnership in combination with the early adopter. Who will welcome and introduce the partnership will be determined. Ideally, the early adopter will welcome and introduce one member of the academic or community partnership, at which time that member will introduce the rest of the team. During the informational meeting, an overview of how and why the partnership was developed and who the members of the partnership are, as well as of the funding, deliverables, and how each county was selected, will be provided. National and regional overdose and PHVM data will be shared. An explanation of what community-based participatory approaches are and why they will be used in this project will be provided, as well as a description of how interviewers will be hired and trained. The findings from each county’s interviews will be shared after interviews have been completed and analyzed. This information will assist in determining the optimal placement of the PHVMs within each community.

In addition, the findings will guide the next steps within each community. The partnership will collaboratively identify additional individuals or organizations to include in the implementation or sustainability of the PHVMs. The partnership and community decision-makers will develop a sustainability plan for the PHVMs based on the findings and each community’s unique needs. The sustainability plan will vary depending on the county’s resources and support and the items to be placed in the PHVMs. For example, if the interview results reveal that people with lived experience report that they would like to have syringes in the vending machine but the decision-makers are not in support of such an item, then a decision will be made based on the communities’ feedback and alignment with current legal and health policies. This determination will be made based on the community members, not the partnership; this promotes buy-in and ownership over the PHVMs, both of which are important for a successful implementation and sustainable project. The community will be informed that naloxone is the only item required in the PHVM due to being a deliverable in the grant. The brand of naloxone (eg, Narcan or RiVive) and how it is administered (eg, nasal or intramuscular) is negotiable. The development of the sustainability plan may include the identification of grants, local or state funding, grassroots and faith-based organizations, and other social and health agencies.

## Results

Data will be collected from 2024 to 2025. Five PHVMs will be implemented in 5 rural NC counties. The location and supplies of the PHVMs will be determined based on stakeholder interviews and community collaborations (eg, public health director; members of the emergency medical services, sheriff, and fire departments; hospital administrators; faith-based organizations; county commissioners; town managers; and prevention and harm reduction advocates).

**Figure 8:**
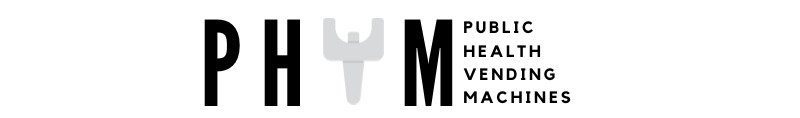
Public health vending machine logo.

The results of this study protocol will be the development of a PHVM implementation toolkit designed to offer practical materials such as email templates, guides, and resources to support other communities in implementing a PHVM (Figure 8). An implementation framework will be used to develop an implementation plan with the community. The exact factors related to the implementation and placement of the PHVMs will be shared in future manuscripts.

Funding for this project was provided by the Health Resources and Services Administration of the US Department of Health and Human Services, from August 2023 to August 2025. Institutional review board approval for this project was received from September 11, 2023, to May 31, 2026. Interview data collection occurred from June to December, 2024. Data analysis for the interview data has been completed, with one publication under review in August 2025. The sustainability data for the PHVM is currently being collected with the anticipation of one publication being produced in 2026.

## Discussion

### Anticipated Findings

This study will be designed to engage a community-based participatory approach to improve naloxone access in rural communities. Interviews with local community members with lived experience, county leadership, and other community decision-makers will be used to examine the optimal placement for implementing a PHVM stocked with naloxone. In addition, community partners will assist the academic team in the development of a sustainability plan for each county.

A key innovation of this study design will be the training of individuals with lived experience from each rural community to serve as recruiters and interviewers. This approach will not only foster trust and rapport with participants but also ensure that the voices of individuals who are often marginalized or unheard are meaningfully included. By empowering community members to lead data collection, we will facilitate the authentic representation of local perspectives and will create a direct line of communication between these voices and community decision-makers.

### Limitations

The proposed study will use a CBPR approach focused on 5 rural counties in NC, which imposes some limitations on the types of data and the nature of the information that can be collected. We expect that additional limitations may be identified during study conduct. The results-oriented manuscript will identify and describe new limitations to follow the PHVM intervention.

## Appendix

Multimedia Appendix 1 Interview guide—page 1.

Multimedia Appendix 2 Interview guide—page 2.

Multimedia Appendix 3 Interview guide—page 3.

Multimedia Appendix 4 Recruitment script.

## Abbreviations

CBPR: community-based participatory research
CINC: Community Impact North Carolina
NC: North Carolina
NCHRC: North Carolina Harm Reduction Coalition
PHVM: public health vending machine
SSP: syringe services program

## Data Availability

Data will be available upon reasonable request with the corresponding author. Interview data will be published upon completion. The public health vending machine implementation toolkit will be published upon completion.
