# Supplementary material for: Introducing Public Health Vending Machines in Rural Communities: Protocol for a Study Using a Community-Based Participatory Approach
Source: JMIR Res Protoc. 2025 Sep 17;14:e64913. doi: 10.2196/64913 (PMC12489422; doi:10.2196/64913)
Supplement: Multimedia Appendix 1 [file resprot_v14i1e64913_app1.pdf]

Supplement 1.0 Interview Script

Bolded sections are the questions you will ask the interviewee
<u>Underlined</u> sections are the instructions for the interviewer
Italicized sections are sections that you will read to the interviewee that provides them information

OBTAIN CONSENT FROM THE PARTICIPANT

<u>At the conclusion of obtaining verbal consent inform the participant that you are going to start recording.</u>

<u>Turn on the recorder</u>

(state the alias) do you agree to participate in this study?

If they say no, say **thank you** and <u>conclude the interview and turn off the recorder</u>.
If they say yes, <u>proceed to the interview</u>.

Thank you for agreeing to be interviewed.

The first few questions are your demographic information.
Can you please state your
1) Age
2) Gender
3) Race/Ethnicity

Thank you (alias)

START INTERVIEW

As a reminder the purpose of our study is to improve the health and reduce deaths of people who use substances in (state county name) county. Our plan is to do this by developing a site where the community members will be able to access naloxone 24 hours a day without having to interact with another person. I will be asking you a few questions that will help us determine what type of naloxone distribution site will be best and where the site should be in your community.
Tell me about how people obtain naloxone in your community?

Questions concerning experience obtaining naloxone in the community –

Who do you feel needs access to naloxone?
Where do people obtain naloxone in your community?

When do you believe people access naloxone?

Have you had experience obtaining naloxone at a pharmacy? Tell me about that experience?

Questions concerning accessing naloxone at a pharmacy –

If they have obtained naloxone from a pharmacy: **Would you obtain naloxone again from a pharmacy? Why or why not?**

If they have not obtained naloxone from a pharmacy: **Why haven't you obtained naloxone from a pharmacy?**

What are the challenges of obtaining naloxone in your community?

Questions on obtaining naloxone –

Is naloxone available when people need or want it? <u>If the person says no, ask the following question:</u> **Please tell me more about that.**

Do you believe people in your community would prefer to access naloxone in a private or public environment? Why?

Is cost a concern?

Do you believe people in your community experience any stigma obtaining naloxone? <u>If the person response with a single response of "yes" or "no" then ask the following probing question:</u> **Please tell me more about why you believe that?**

Do you believe that your community members know what naloxone is and why it is used?

Our goal is to provide naloxone in the community where individuals can access it at any time of day without talking or interacting with another person. The distribution options we are offering will provide a selection of health-promoting items, such as naloxone, hygiene products, and informational pamphlets. I am going to show you some options that are being considered to distribute naloxone in your community.

<u>Show photos of options</u> (which items would be considered) for naloxone distribution. Read the following to the participant and point to each option as you read.

Option 1 is a public health vending machine. A public health vending machine is a convenient and accessible and has an automated dispenser. The machine can hold approximately 140 items.

What are your thoughts on this option for your community?

Option 2 is a wall-mounted vending machine. A wall-mounted vending machine is designed to take up limited space and can be mounted in public restrooms or lobbies. The machine holds approximately 50 items.

What are your thoughts on this option for your community?

Option 3 is a free-standing outdoor distribution stand. This stand is moveable and is designed after a newspaper stand. These stands are usually places in high density areas such as malls, parks, etc. The stand can hold approximately 20 items.

Option 4 is a pole mounted distribution box. This box is mounted to a pole in the community. These boxes are mounting in outdoor locations. They can be mounted on small to large poles. This box holds approximately 6 items.

What are your thoughts on this option for your community?

Out of all the options we discussed what do you believe would be the best option for naloxone distribution in your community? Why do you feel that way?

Besides naloxone, are there other items that you believe would be helpful to be dispensed from the distribution options we just discussed?

Where would the ideal location(s) be in your community to place the (state the option the person picked from the above question).

How might we promote/spread the word about this option (distribution of naloxone) to people who need it?

Do you have anything else you would like to share with us?

Thank you for participating.

Provide them with the gift card and have them verbally state “I received the gift card.”

Turn off the recorder
