# Supplementary material for: Introducing Public Health Vending Machines in Rural Communities: Protocol for a Study Using a Community-Based Participatory Approach
Source: JMIR Res Protoc. 2025 Sep 17;14:e64913. doi: 10.2196/64913 (PMC12489422; doi:10.2196/64913)
Supplement: Multimedia Appendix 2 [file resprot_v14i1e64913_app2.pdf]

When do you believe people access naloxone?

Have you had experience obtaining naloxone at a pharmacy? Tell me about that experience?

Questions concerning accessing naloxone at a pharmacy –

If they have obtained naloxone from a pharmacy: **Would you obtain naloxone again from a pharmacy? Why or why not?**

If they have not obtained naloxone from a pharmacy: **Why haven't you obtained naloxone from a pharmacy?**

What are the challenges of obtaining naloxone in your community?

Questions on obtaining naloxone –

Is naloxone available when people need or want it? <u>If the person says no, ask the following question:</u> **Please tell me more about that.**

Do you believe people in your community would prefer to access naloxone in a private or public environment? Why?

Is cost a concern?

Do you believe people in your community experience any stigma obtaining naloxone? <u>If the person response with a single response of "yes" or "no" then ask the following probing question:</u> **Please tell me more about why you believe that?**

Do you believe that your community members know what naloxone is and why it is used?

Our goal is to provide naloxone in the community where individuals can access it at any time of day without talking or interacting with another person. The distribution options we are offering will provide a selection of health-promoting items, such as naloxone, hygiene products, and informational pamphlets. I am going to show you some options that are being considered to distribute naloxone in your community.

<u>Show photos of options</u> (which items would be considered) for naloxone distribution. Read the following to the participant and point to each option as you read.

Option 1 is a public health vending machine. A public health vending machine is a convenient and accessible and has an automated dispenser. The machine can hold approximately 140 items.

What are your thoughts on this option for your community?

Option 2 is a wall-mounted vending machine. A wall-mounted vending machine is designed to take up limited space and can be mounted in public restrooms or lobbies. The machine holds approximately 50 items.

What are your thoughts on this option for your community?
