# Supplementary material for: Introducing Public Health Vending Machines in Rural Communities: Protocol for a Study Using a Community-Based Participatory Approach
Source: JMIR Res Protoc. 2025 Sep 17;14:e64913. doi: 10.2196/64913 (PMC12489422; doi:10.2196/64913)
Supplement: Multimedia Appendix 3 [file resprot_v14i1e64913_app3.pdf]

Option 3 is a free-standing outdoor distribution stand. This stand is moveable and is designed after a newspaper stand. These stands are usually places in high density areas such as malls, parks, etc. The stand can hold approximately 20 items.

Option 4 is a pole mounted distribution box. This box is mounted to a pole in the community. These boxes are mounting in outdoor locations. They can be mounted on small to large poles. This box holds approximately 6 items.

What are your thoughts on this option for your community?

Out of all the options we discussed what do you believe would be the best option for naloxone distribution in your community? Why do you feel that way?

Besides naloxone, are there other items that you believe would be helpful to be dispensed from the distribution options we just discussed?

Where would the ideal location(s) be in your community to place the (state the option the person picked from the above question).

How might we promote/spread the word about this option (distribution of naloxone) to people who need it?

Do you have anything else you would like to share with us?

Thank you for participating.

<u>Provide them with the gift card and have them verbally state "I received the gift card."</u>

<u>Turn off the recorder</u>
