# Supplementary material for: Introducing Public Health Vending Machines in Rural Communities: Protocol for a Study Using a Community-Based Participatory Approach
Source: JMIR Res Protoc. 2025 Sep 17;14:e64913. doi: 10.2196/64913 (PMC12489422; doi:10.2196/64913)
Supplement: Multimedia Appendix 4 [file resprot_v14i1e64913_app4.pdf]

Recruitment Script

Hello

I am helping conduct interviews for a research study to reduce overdose deaths among people who use substances in North Carolina by increasing access and availability to naloxone. We are looking for participants to be interviewed to gain insight into naloxone access in [Carteret, Jackson, Stanly, Surry, or Swain] county

Participation will include an interview that will last about 60 minutes. All participants who agree will be assigned an alias for privacy. Participants will be compensated with a $30 gift card.

To be eligible to participate you must be:
18 years or older
Live in [Carteret, Jackson, Stanly, Surry, or Swain] county
Have used one of the following in the last 2 years: opioids, fentanyl, cocaine, psychostimulants

Would you be interested in participating in this interview?

If no,
Thank you for your time and consideration.

If yes,
Thank you for being willing to participate.

If you know of anyone that might be interested in participating, please give them one of these cards. Provide them with 5 cards.
